# Cultivating Safety: How Food Safety Champions Translate Regulatory Compliance into Frontline Practice

**DOI:** 10.3390/foods15142466

**Published:** 2026-07-12

**Authors:** Xiaochen Liu, Phil Bremer, Miranda Mirosa

**Affiliations:** 1 Department of Food Science, University of Otago, Dunedin 9054, New Zealand; xiaochen.liu@otago.ac.nz (X.L.); phil.bremer@otago.ac.nz (P.B.); 2 New Zealand Food Safety Science and Research Centre (NZFSSRC), Palmerston North 4442, New Zealand

**Keywords:** food safety culture, institutional theory, practice enactment, organisational behaviour, behavioural reinforcement, safety governance, implementation mechanisms

## Abstract

This Perspective examines a central problem in food safety governance: why organisations with well-developed food safety management systems, standard operating procedures, and audit mechanisms may still struggle to achieve stable and consistent frontline food safety practices. Using a narrative integrative review approach, this paper draws on the literature on food safety culture, organisational behaviour, institutional theory, implementation Champions, and frontline practice. It argues that the food safety is not only dependent on the effectiveness of the formal food safety systems, but also on how institutional expectations are interpreted, negotiated, reinforced, and enacted in everyday work. This paper introduces the concept of Food Safety Champions and conceptualises them as translational actors who can help connect formal food safety requirements with situated practice through meaning making, contextual negotiation and behavioural reinforcement. It further suggests that a Champion’s effectiveness depends not only on individual initiative, but also on peer trust, organisational legitimacy, their informal networks and connections, enabling conditions, and continuous institutional feedback. This paper contributes a practice-based conceptual framework for understanding how formal food safety systems may become more meaningful, sustainable, and behaviourally effective in food production environments.

## 1. Introduction

Contemporary food safety governance relies heavily on formal institutional systems. Structured frameworks centred on Food Safety Management Systems (FSMS), Hazard Analysis and Critical Control Points (HACCPs), and Standard Operating Procedures (SOPs), supported by audit and verification mechanisms, provide the institutional foundation of organisational risk management and compliance [1,2]. These systems define expected standards of action and provide operational certainty across global food supply chains. However, the presence of formal systems does not automatically ensure stable and consistent frontline food safety practices. Despite increasingly sophisticated regulatory requirements and expanding audit regimes, the global burden of foodborne disease remains persistently high [3]. Organisations continue to invest in formal control systems, increasing both their complexity and coverage [4], yet technically driven and compliance-oriented governance approaches do not always produce the expected food safety outcomes.

This tension highlights a central challenge at the centre of food safety governance: formal systems can specify what should happen, but they cannot fully control how employees act under everyday production conditions. Frontline decisions are often shaped by time pressures, production targets, labour shortages, operational disruptions, changes to team routines, local judgement, and peer norms. A persistent gap may therefore emerge between the behaviours prescribed by formal institutions and the behaviours enacted under everyday frontline conditions. From an organisational perspective, this reflects a form of decoupling between institutional logic and practical logic, or between formal structures and everyday actions [5,6,7]. In audit-driven environments, this problem may appear as means–ends decoupling, where organisations maintain training, audits, and compliance documentation, yet these formal activities do not consistently translate into safer outcomes [4,6].

Research on Food Safety Culture (FSC) has increasingly shifted attention from technical compliance alone towards the social and behavioural conditions that shape everyday practice [7,8,9,10,11]. Existing studies have identified leadership, communication, and resource allocation as important factors influencing food safety behaviour [12,13,14,15]. However, a key question remains: why do teams operating under the same formal system often demonstrate different levels of execution and consistency? This observation suggests that the movement from institutional expectation to everyday practice requires more than the existence of written procedures or audit mechanisms.

Within this context, the Food Safety Champion provides a useful entry point for examining how formal expectations may become meaningful and actionable in frontline work. Industry and regulatory guidance increasingly emphasise that food safety is sustained not only by technical controls and documentation, but also through visible role modelling, ongoing feedback, and a shared sense of responsibility [16,17,18]. Existing studies commonly describe Champions as influencers characterised by their informal responsibilities, willingness to lead by example and cross-functional coordinating roles [18]. Despite this recognition, Champions remain under-theorised in the academic food safety literature and are often discussed within conventional leadership or behavioural intervention frameworks [4,14,19].

This paper introduces the concept of Food Safety Champions, hereafter referred to as Champions, as translational actors who play a role in connecting institutional expectations with everyday practice, through their practical knowledge and experience, modelling of good behaviours (behavioural reinforcement), and connections. Hence, Champions can support the interpretation of abstract requirements within local working conditions and help reinforce food safety behaviours over time. This does not imply that Champions can replace formal systems or operate independently of organisational support. Rather, their effectiveness depends on them working with organisational legitimacy, connections, and continuous support and feedback.

This paper addresses four interrelated questions. First, how can the Food Safety Champion be conceptualised beyond the idea of a proactive employee or informal leader? Second, how may Champions move institutional expectations into everyday work through meaning making, contextual negotiation, and behavioural reinforcement? Third, what supporting and enabling conditions sustain the effectiveness of the Champion’s role? Fourth, how can frontline practice generate feedback that enables companies to adapt formal systems and reduce the gap (decoupling) between formal structures and everyday action?

**Figure 1:**
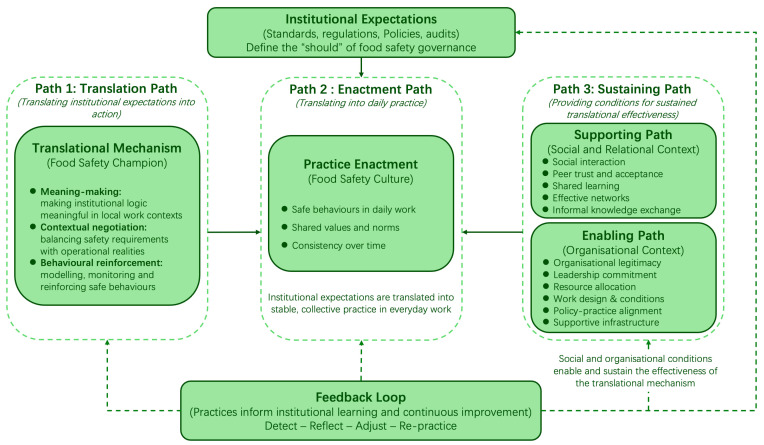
The Translation–Enactment–Sustaining (TES) Food Safety Framework for Food Safety Champions, showing three interrelated pathways and a feedback loop.

By integrating the literature on food safety culture, human factors, organisational behaviour and institutional theory, this paper develops a practice-based conceptual framework built around institutional expectations, translational mechanisms, practice enactment, and feedback loops. For clarity, this food safety-specific conceptual framework is referred to here as the Translation–Enactment–Sustaining (TES) Food Safety Framework. As shown in Figure 1, the framework contains three interrelated pathways: the translation path (Path 1), which explains how institutional expectations may be interpreted and turned into action; the enactment path (Path 2), which shows how translated expectations become collective daily practice; and the sustaining path (Path 3), which examines how peer trust, effective networks, and organisational enabling conditions support the long-term effectiveness of the Champion role. Through this framework, the paper contributes a conceptual explanation for how formal food safety systems may become more closely connected with everyday frontline practice.

## 2. Cross-Sector Perspectives: Theoretical Foundations of the Food Safety Champion

To ensure transparency in the development of this Perspective, the conceptual synthesis was informed by scoping review principles and a concept-driven source selection strategy [20,21,22]. Because the purpose of the paper was conceptual synthesis rather than exhaustive evidence mapping, the synthesis process was designed to identify, delimit, and integrate the literature streams relevant to the development of the Food Safety Champion framework. As the manuscript was not intended to be a systematic review or a PRISMA-ScR scoping review, a formal PRISMA flow design was not used. Instead, transparency was ensured through an explicit description of the information sources, search streams, eligibility logic, charting categories, and synthesis procedure. Narrative synthesis principles were used to integrate conceptually heterogeneous sources and to identify recurring mechanisms across food safety culture, organisational behaviour, institutional theory, and the implementation literature [23].

The literature was included when it addressed food safety culture, Champion or Champion-like roles, behavioural or organisational mechanisms, peer-based networks, or the translation of formal requirements into everyday practice. Sources were excluded if they: focused only on technical compliance or regulatory requirements without discussing behavioural or organisational mechanisms; lacked traceable publication information; or had limited relevance to food safety or transferable organisational practice. The included sources were organised according to bibliographic information, source type, sectoral context, key concepts, Champion-related activities, functional mechanisms, enabling conditions, and transferability to industrial food settings. Initial open coding was followed by thematic clustering, allowing the literature to be organised into the functional streams presented in Table 1. The peer-reviewed literature was used primarily to support the conceptual and theoretical argument, while grey literature was used as supplementary evidence of current industry practice and implementation guidance, rather than as definitive evidence of intervention efficacy.

Table 1 therefore presents the literature base not as a complete systematic evidence map, but as a conceptual classification of the main literature streams used to develop the Food Safety Champion framework.

Table 1 illustrates how cross-sector evidence on a Champion’s roles, behavioural reinforcement, peer-based practice support, and organisational implementation as described in the scientific literature was used to inform the Food Safety Champion framework. Champions have consistently emerged to bridge the gap between formal institutional frameworks and sustained behavioural change. This critical translation role has proven to be vital across diverse domains, including organisational management, healthcare compliance, corporate sustainability, workplace wellness, and community-based interventions [27,28,29,31,33]. Recent cross-sector research further suggests that a Champion’s effectiveness depends not only on individual enthusiasm, but also on role legitimacy, organisational resources, peer influence, and enabling conditions [31,33].

Building on the literature streams summarised in Table 1 and the literature on cross-sector Champions, the ways in which the role of the proposed Food Safety Champion construct differs from other related organisational and implementation roles is outlined in Table 2. Although Food Safety Champions share some features with informal leaders, change agents, opinion leaders, Safety Champions, boundary spanners, and food safety ambassadors, the construct proposed in this paper is more specifically positioned within food safety governance. It refers to actors who help translate formal food safety expectations into situated frontline practice through meaning making, contextual negotiation, behavioural reinforcement, peer-based support, and feedback to the formal system.

This distinction between the constructs and their functions outlined in Table 2 is important because the value of Food Safety Champions lies not only in their influence as individuals, but in their translational position between formal food safety systems, frontline practice, and organisational learning.

Research shows that the influence of Champions rarely depends on formal authority alone. Their value lies in their ability to help organisational goals become understood, accepted, and sustained in practice. Solitander et al. [29] argue that the continuity of organisational change depends not only on the adoption of a formal strategy, but also on key individuals who interpret goals, connect resources, and maintain direction in everyday work. May [30] further emphasises that Champions are not simply implementers, but actors who continuously translate between institutional logic and local practice.

This function is equally visible in sustainability and health promotion. Pekaar et al. [31] found that Champions facilitate the internalisation of practice through relational networks and cross-functional coordination. Alm and Hultman [32] similarly show that a Champion’s effectiveness depends more on peer trust and practical credibility than on formal authorization.

Taken together, these studies suggest that formal systems often become decontextualised when translated into individual behaviour. Institutions can define goals and procedures, but they cannot automatically explain how these expectations should be understood and enacted in specific working conditions [30,31]. Champions can help bridge this gap through providing explanations, coordination, role modelling, and visible demonstration, allowing institutional expectations to enter local practice in more meaningful and sustainable ways [31,32,33].

For this reason, introducing the Champion concept into food safety is not a simple transfer of terminology, but the application of a transferable mechanism, as food safety faces the same tension between written rules and operational reality as other organisational areas [8,18]. Recent research has emphasised that FSCs should be understood as a behavioural and organisational phenomenon shaped by maturity, leadership, employee participation, and the internalisation of safe practices, rather than only by formal documentation or audit compliance [9,10]. Champions are therefore relevant because FSC depends not only on what is written, but also on how food safety expectations are made meaningful and sustainable in everyday work practices.

One important mechanism through which Champions influence behaviour is meaning making. For management, institutional requirements are often clear and necessary. For frontline employees, however, the central question is, what do these rules actually mean, in the context of their everyday work. When food safety requirements are understood only as externally imposed tasks, compliance tends to remain passive, and behaviour may be driven by audit requirements rather than genuine risk awareness.

The recent literature supports this behavioural interpretation by showing that FSC maturity involves the movement from compliance-oriented control towards behavioural internalisation and shared responsibility [9,10]. This is directly relevant to the Champions role, as they can help make abstract food safety requirements understandable within the practical language, pressures, and routines of frontline work.

A second mechanism by which Champions can encourage good food safety practices is through behavioural reinforcement. Understanding the rules does not guarantee that safe behaviours will be sustained. Training communicates what should happen, but everyday behaviour is shaped more powerfully by what is actually tolerated and what is consistently upheld in practice. Employees learn organisational boundaries by observing which deviations are ignored and which standards are non-negotiable [5,8,18]. FSC is therefore sustained less by formal training and more by whether correct behaviour remains visible and repeatedly reinforced.

Champions play a critical role in behavioural reinforcement through continuous modelling and immediate feedback. Demonstrating the importance of maintaining hygienic practices during production peaks, correcting deviations at critical control points, and encouraging reporting when problems arise, are more influential prompts for good food safety practices than one-off training sessions [27,36]. Recent empirical work on FSC interventions also suggests that organisational maturity can be strengthened when behavioural expectations are actively supported, monitored, and reinforced in practice [10]. More importantly, they may help prevent the normalisation of deviance. As Reason [34] argues, many systemic failures do not begin with major errors, but with small violations that are repeatedly tolerated until they become accepted as normal practice. The sustained visibility and actions of a Champion can help interrupt this process by making safe behaviour observable, socially supported, and repeatedly reinforced under real operational conditions.

A third mechanism by which Champions can encourage good food safety practices is associated with relational trust and insider legitimacy. The influence of a Champion is rarely grounded in formal authority; rather, it is built primarily on relational trust. Employees often maintain a natural distance from management-imposed requirements but are far more likely to respond to colleagues who understand operational pressures and are consistently present during everyday work [31,32,37].

Alm and Hultman [32] argue that the effectiveness of a Champion depends heavily on their social legitimacy. Employees accept their guidance not because they represent the formal system, but because they are perceived as people who genuinely understand frontline realities. This insider status gives their actions greater credibility and enables food safety expectations to move from managerial expectations into shared team practice. Recent cross-sector Champion studies similarly emphasise that credibility, belonging, and multi-level support shape whether Champions can influence behaviour beyond formal instruction [27,29]. Whether institutional requirements truly enter practice depends partly on the level of trust employees have in the people bringing the requirements to their attention.

The core of FSC lies not in the existence of formal systems, but in how employees make choices under production pressure and resource constraints. Jespersen et al. [18] argue that the key role of the Food Safety Champion is to maintain the visibility and priority of food safety, ensuring that safety requirements are not displaced by efficiency pressures. The introduction of behaviour-oriented indicators in BRCGS, together with FSANZ’s emphasis on employee participation and leadership, demonstrates the importance that these organisations place on FSCs and reflect that it is increasingly being understood as a process enacted through people, routines, and everyday behaviour, rather than as the automatic outcome of written systems [9,10,16,17].

For this reason, Food Safety Champions should not be understood merely as proactive employees or informal leaders, but as a core translational mechanism between institutional expectations and practice enactment. This does not mean that Champions alone can resolve food safety culture challenges. Rather, their effectiveness is dependent on organisational recognition, peer trust, their practical credibility and the enabling resources, and wider support structures that allow safe behaviour to be understood, reinforced, and sustained over time [10,31,33].

## 3. Institutional Expectations: Why Formal Systems Require Translation

Formal institutions can define what should happen, but they cannot directly determine what people actually do in practice. Food safety management systems, HACCP, and SOPs establish what employees are expected to do by specifying responsibilities, critical control points, and operational standards [1,2]. They provide direction, but they cannot replace judgement in practice.

The central challenge of food safety governance lies in how these rules enter everyday work. Hence, formal systems provide the normative standard, but culture is formed in the practical decisions made under real working conditions.

Food production is constantly shaped by time pressures, production targets, labour constraints, and equipment variability. Employees are rarely making a simple choice about whether to follow a rule; rather, they are frequently required to make continuous judgements about competing priorities, efficiency, safety, and operational reality. Decisions about stopping production, managing contamination risks, maintaining line continuity, completing cleaning procedures, or reporting deviations are not made during audits, but in everyday practice beyond the written procedures.

Many safety deviations do not arise from deliberate actions, but from ongoing adaptation to system pressures and practical constraints [34]. Food safety behaviour is shaped not only by knowledge, but also by workload, risk perception, local routines, communication patterns, and peer norms [14,39]. Institutions may define standards, but they cannot on their own determine their employee’s actions. For example, an organisation may possess complete documentation, deliver training, and pass audits, yet still experience repeated deviations in frontline practice. This observation highlights a fundamental distinction between formal compliance and practice enactment. The former demonstrates that an organisation has a system; the latter determines whether the system actually works. The problem in food safety governance is often not the absence of formal structures, but the assumption that formal structures can enter practice on their own [5,6].

This challenge has increasingly been recognised across the food industry. BRCGS has incorporated behaviour-oriented indicators into its evaluation framework, treating leadership demonstration, employee engagement, and consistency of everyday behaviour as core evidence of system effectiveness, rather than focusing solely on the completeness of formal documentation [16]. The Alliance to Stop Foodborne Illness and FSANZ make a similar point: food safety culture is not a static institutional condition, but a dynamic process continuously shaped through communication, visible examples, and shared responsibilities [17,38]. A mature food safety system is not defined by how complete its procedures are, but by whether critical controls are upheld under pressure.

Institutions, therefore, require more than simple transmission of the rules, they require the rules to be translated into everyday practice. This is the theoretical foundation of the Food Safety Champion, as they connect institutional expectations with practice enactment, giving abstract rules practical meaning and preventing food safety requirements from being diluted by efficiency pressures. Without such mediating processes, formal expectations may remain weakly connected to everyday practice. Translational mechanisms therefore help explain how documented requirements may become incorporated into routine behaviour.

In this paper, translation is not used to describe the simple transmission of information from management to workers. Rather, it refers to the institutional process through which formal rules, standards, and expectations are interpreted, adapted, edited, and re-embedded within local practice. The institutional translation literature, particularly within Scandinavian institutionalism, emphasises that organisational ideas and rules do not move unchanged across contexts, but are translated and edited as actors make them meaningful within specific organisational settings [43,44,45]. This perspective aligns with institutional work theory, which highlights how actors maintain, repair, or reshape institutions through practical action [46]. Food Safety Champions can therefore be understood as actors who perform institutional translation work by helping formal food safety expectations become intelligible, workable, socially reinforced, and responsive to feedback within frontline production environments.

This institutional translation perspective is particularly important in food production environments, where formal requirements often concern risks that are not immediately visible at the point of action. HACCP plans, SOPs, audit criteria, and certification requirements define expected controls, but these controls only become effective when they are interpreted and enacted consistently during routine production. Food safety therefore creates a distinctive translation problem: formal rules must be made meaningful within work settings shaped by time pressure, labour constraints, operational variations, and competing production priorities [1,2,16,17,18,38].

## 4. Translational Mechanism: How Food Safety Champions Translate Institutional Expectations into Practice

Institutions define what should happen, while Food Safety Champions help turn these expectations into what is actually done in complex production environments. Because formal systems cannot fully anticipate the dynamic pressures of frontline work, such as time pressure, labour shortages, or equipment variability, everyday decisions often depend on immediate risk interpretation and the behavioural norms recognised within teams [18,34].

In practical terms, this translational work is visible when Champions help teams decide how formal food safety expectations should be upheld during specific operational situations. These may include cleaning verification, temperature control, allergen segregation, cross-contamination prevention, labelling checks, deviation reporting, or decisions about whether production should stop or continue. These examples illustrate why Food Safety Champions are not simply general implementation supporters, but actors whose role is tied to the everyday enactment of food safety management systems under real production pressures [1,2,10,16,17,18,38]. In this context, the value of the Champion does not come from formal authority, but from their position as a translational mechanism between institutional expectations and frontline action. Through meaning making, contextual negotiation, and behavioural reinforcement, Champions may help translate macro-level governance goals into more stable micro-level practice. This is not simply the transmission of information, but the ongoing enactment of institutional logic in everyday work [30,31].

Champions can support meaning making by helping turn rules from external requirements into practical understanding. Institutional expectations are usually expressed through standards, procedures, and audit indicators. For management, these rules may appear as clear instructions, while for frontline employees, the key challenge is how to connect these abstract requirements with the realities of their own work. When food safety is perceived only as an additional administrative burden, organisations tend to remain at the level of passive compliance.

Champions can help make institutional requirements move beyond administrative language by reframing the risk in practical terms, for example cross-contamination control is not seen solely as an audit requirement, but as a way of protecting consumers, and deviation reporting becomes not merely a procedural step, but a defence against system failure [10]. This cognitive shift allows institutional expectations to move from external compliance pressure to the use of employees’ own practical judgement. Safety standards are then more likely to be understood as principles worth actively choosing and maintaining in daily work [30,35].

Champions can contribute to contextual negotiation by helping make formal requirements work under real production conditions. Understanding a rule does not mean that it is automatically workable. Food production often places institutional logic into direct conflict with efficiency demands. When peak production coincides with the need for a full cleaning procedure, employees are not facing a technical problem, but a judgement about priorities.

In such circumstances, the Champion acts as a contextual negotiator. Their role is not to demand mechanical compliance, but to make institutions workable without compromising critical principles. Through on-site coordination, practical judgement, and cross-level communication, they help rules function within real operational constraints.

Many safety deviations do not result from an unwillingness to follow rules, but from behaviours shaped by system pressure and practical limitations [35]. Food safety management must therefore focus on how rules are enacted in real production environments [7]. Champions can help teams determine which controls remain non-negotiable when operational pressures compete with food safety priorities [31].

As discussed earlier, Champions can provide behavioural reinforcement by helping make safe behaviour visible, repeated, and socially supported. Food safety culture is not built through one-off training, but through repeated practice over time [8,47,48]. Employees continuously read the real signals within a team, such as which deviations are tolerated, which standards are consistently upheld, or whether critical controls remain visible during periods of pressure. Long periods without visible incidents can weaken organisational sensitivity to risk, allowing deviations to become gradually normalised and complacency to develop [40].

At this stage, Champions contribute through their continuous modelling of good behaviour and timely feedback. This may involve insisting on visible cleaning during production peaks, escalating issues when labelling or temperature checks are inconsistent, or ensuring that unresolved risks are discussed openly rather than quietly absorbed into the routine. What matters here is not their formal authority, but their visibility and consistency. Repeated signals shape what teams come to treat as normal [10,34]. In this way, Champions help transform institutional expectations into stable behavioural routines.

The functional positioning of the Champion is therefore important. Existing studies often describe Champions as proactive employees with strong responsibility and leadership qualities [49,50], but this is not sufficient to explain their distinctive role between institutions and practice. Their value lies not only in their personal traits, but also in their position between institutional logic and frontline reality. Hence, their effectiveness depends on them having dual credibility: upward, in communicating operational constraints to management, and downward, in helping teams understand why certain food safety requirements remain non-negotiable in practice. Without this legitimacy, formal systems are more likely to be perceived as external demands rather than shared responsibilities.

Champions are therefore best understood not simply as informal leaders, but as translational actors who help sustain the connection between institutional expectations and everyday practice. This positioning does not imply that Champions alone can guarantee cultural change. Rather, it suggests that their effectiveness depends on the quality of translation and the organisational conditions that support it.

Translation should therefore be understood as a condition that can strengthen, rather than replace, formal food safety systems. Without effective translation, institutions remain at the level of documents, audits, and symbolic compliance. Even when systems appear complete, behaviour can remain unstable if institutional expectations are not meaningfully connected to everyday decisions, team interactions, and organisational routines.

The sustained work of Champions may help institutional expectations enter everyday practice, but this depends not only on individual initiative. It also depends on the organisational design, motivational structures, cross-functional collaborations, and peer networks that provide practical support [30,31,32,35,51]. In community settings, community Champions sustain collective practice and system resilience through identity, belonging, and peer relationships [37].

## 5. Practice Enactment: How Food Safety Culture Becomes Visible

An FSC becomes visible not only through policy statements, training records, or audit preparation, but through the small and repeated decisions people make during ordinary work. Employees constantly observe what is accepted in practice, such as whether hygiene shortcuts are tolerated, whether contamination risks are escalated, and whether managers respond seriously when concerns are raised.

Culture is reflected more clearly in shared behavioural patterns than in formal declarations [8]. The real test of an FSC is how people behave when no one is watching [47,48]. Food safety culture can therefore be understood not simply as an organisational statement, but as the consistency of behaviour repeated over time.

Shared behavioural norms are central to this process as an FSC is most clearly expressed through the behavioural norms that teams treat as legitimate in everyday work. New employees quickly learn these norms by observing what is tolerated, rewarded, questioned, or ignored. They notice whether stopping production because of a contamination risk is treated as being a responsible action or as an unnecessary disruption, and whether reporting a deviation is seen as professionalism or as creating trouble.

The stability of an FSC should not be judged by isolated examples of correct behaviour, but rather by the consistency of collective practice across people, shifts, and operating conditions. When hidden rewards continue to favour speed, silence, or convenience over control, institutional logic is weakened in practice [7]. A mature FSC therefore depends on whether safe behaviour remains socially supported, operationally possible, and consistently enacted, rather than only stated as an organisational value.

How workers behave under production pressures is especially important because it reveals how safety priorities are interpreted under real operational conditions. A company’s FSC becomes particularly visible when production pressures compete with safety priorities. Under conditions such as equipment failure, staffing shortages, or time pressure, the practical strength of a food safety system is reflected in whether critical controls remain protected, procedures are completed appropriately, and reporting is supported rather than discouraged.

Reason [34] argues that safety is not proven under ideal conditions, but when systems are under strain. Long periods without visible incidents can also create complacency, gradually reducing sensitivity to emerging risks [36,37]. A mature system is therefore not one without problems, but one in which critical controls remain non-negotiable even under pressure.

An FSC is also reflected in how organisations communicate about risk. Whether risks are identified and addressed in a timely manner depends largely on whether employees feel psychologically safe enough to raise concerns, admit mistakes, and report small deviations before they become larger failures. When reporting a problem is expected to result in blame, punishment, or damaged relationships, reporting is often suppressed. In such cases, surface stability can conceal deeper vulnerabilities.

The most dangerous systems are not necessarily those with many visible problems, but those in which problems are no longer being made visible. By contrast, open communication allows small deviations to be identified before they develop into serious failures [38]. FSANZ emphasises that leadership demonstration and employee participation are not secondary conditions, but core mechanisms through which culture is sustained [17]. A mature system is therefore one in which problems remain visible and are taken seriously.

Institutions provide direction, but practice enactment determines whether those expectations become real outcomes. A good FSC is therefore expressed through repeated operational choices, such as whether cleaning procedures are still followed when no one is watching, whether production is delayed to eliminate a contamination risk, or whether teams continue to protect critical controls when efficiency pressures increase.

Bromley and Powell’s [6] concept of means–ends decoupling reminds us that audit records and training frequency may reflect managerial effort, but they do not necessarily indicate cultural maturity. Hence, formal compliance alone does not demonstrate a mature FSC if everyday practice moves in a different direction. An FSC is not something an organisation possesses, but rather is something it continuously enacts through everyday work.

## 6. Supporting and Enabling Conditions for Sustained Champion Effectiveness

The influence of a Food Safety Champion rarely comes from formal authority; rather, it begins with peer trust. Employees may not necessarily change their behaviour simply because management issues a requirement, but rather they are far more likely to respond to requests by people who are consistently present in their daily work, understand operational pressures, and have long been part of the frontline environment.

This credibility is not granted solely by their position, but built gradually through practical experience, problem-solving ability, and consistent behaviour. Hence, a Champion’s effectiveness depends heavily on their accessibility and trustworthiness [32]. Employees respond to a Champion’s guidance not because they represent the formal system, but because they are seen as people who genuinely understand frontline realities. Without this relational foundation, institutional translation is difficult to sustain.

Networks and connections ensure that critical knowledge is transmitted not only through training and formal procedures, but also through shared practice and continuous interaction [52,53]. In food safety management, decisions about when production must stop, when deviations require immediate escalation, and how efficiency should be balanced with control cannot be fully determined by written procedures alone; rather, they are learned collectively through practice. Within a network Champions do more than pass on information; they help interpret practice. They help new members understand the team’s implicit behavioural rules, allowing food safety to move from individual responsibility to stable collective practice.

At the same time, Champions require support networks if their role is to be sustainable. Many organisations respond to FSC challenges by simply appointing a “motivated person” as a Champion, while neglecting the support systems required to sustain the role. This often leaves Champions carrying significant responsibility without meaningful structural support. When a Champion’s work is treated as an additional duty rather than an embedded function, role fatigue and declining engagement are common [27]. If teams operate with an unspoken rule of “do not create trouble,” or if efficiency is consistently prioritised over safety, the Champion’s influence may gradually weaken.

A Champion may also experience identity tension, as they are expected to protect institutional standards while maintaining peer trust. If they are perceived as an extension of managerial surveillance rather than as a source of peer support, their social legitimacy quickly collapses [32]. If this occurs, they risk being marginalised or reduced to symbols of formal compliance rather than being supported as practical contributors to cultural change. This suggests that the Champion role cannot be sustained by personal motivation alone; rather, it requires a supporting path that protects legitimacy, trust, and continuity.

Organisational enabling is therefore critical to sustain a Champion’s effectiveness. Even with peer trust and networks and connections, a Champion’s influence cannot be sustained without organisational legitimacy, resource commitment, and institutional space.

Pekaar et al. [31] argue that the long-term effectiveness of a Champion depends on whether organisations recognise the value of their role and provide space for communication, coordination, and practical improvement. Champions do not need to be in formal managerial positions, but their function between institutions and practice must be explicitly legitimised.

Leadership demonstration is central to this enabling process. Sustained employee commitment depends largely on whether people believe management genuinely prioritises food safety [16,17]. If formal documents emphasise safety while operational decisions consistently favour efficiency, a Champion’s credibility can quickly erode.

Resource allocation is equally critical. Without sufficient time, staffing, and equipment, food safety can become an unrealistic expectation rather than a workable standard. A mature FSC should not depend on individual persistence alone, but on turning the Champion’s role into organisational capability.

The role of the Champion is also connected to practical recoupling. Standardised procedures can prescribe behaviour, but they cannot generate commitment. Audit systems can detect deviation, but they cannot replace recognition, trust, or a sense of belonging. Employees are not mechanical executors of rules, but rather the stability of their behaviour depends on whether they understand the meaning of their work, feel that their contribution is recognised, and believe that the organisation genuinely values food safety.

For this reason, a Champion’s function can be understood as a means of practical recoupling. Through everyday explanation, peer support, and continuous feedback, Champions can ensure that institutional expectations are linked to human motivation and real working conditions, so that rules become more likely to move beyond paper compliance to becoming practices that people actively choose to maintain.

The purpose of the Supporting Path and the Enabling Path, as shown in Figure 1, is therefore to move the food safety Champion role from being based on individual effort towards being a stable organisational function. The Supporting Path explains why employees continue to treat food safety practices as legitimate and worth maintaining through peer trust, networks and connections, and shared behavioural norms. The Enabling Path addresses why Champions can continue to function, through role legitimacy, leadership commitment, and resource support.

The Supporting and Enabling Paths also explain how organisations may identify and embed Champions in practice. Effective Champions are rarely the most visible people; rather, they are often the person others naturally turn to when risks emerge, judgement becomes uncertain, or standards begin to weaken. The key is to identify individuals who consistently shape everyday practice through their visible actions and influence on team norms.

To improve operational application, this framework has been translated into an industry resource, a Food Safety Champion Support Guide, which provides structured pathways for identification and support [54]. The guide emphasises that Champions emerge through continuous interaction and feedback, rather than from one-off nomination. Their effectiveness depends on cross-level communication, peer trust, and organisational support rather than individual heroism. This further confirms that building a mature FSC is a process of continuous recoupling between institutions, relationships, and practice. Champions cannot simply be appointed; they must be recognised in practice and sustained within the system.

However, a Champion-based approach also has clear limitations. If organisations treat Champions as a substitute for structural support, the role can become dependent on individual enthusiasm, informal authority, and personal resilience [31,33]. This creates a risk of role overload, fatigue, blurred accountability, and discontinuity when key individuals leave or lose influence. The Champion’s peer legitimacy may also weaken if the role is perceived as managerial surveillance rather than one of providing practical support [32]. Further, the Champion’s role may become symbolic, for example if they signal a commitment to FSC but do not appear to have the ability to influence the conditions that shape daily practice [5,6]. For this reason, Champions should not be seen as a low-cost remedy for organisational weaknesses, but as a mechanism that only works when supported by clear role boundaries, protected time, organisational recognition, peer trust, and feedback channels [31,32,33].

A further limitation concerns the TES Food Safety Framework itself. As a conceptual model, the framework necessarily simplifies a dynamic and context-dependent process into a set of pathways and relationships. It identifies how translation, enactment, sustaining conditions, and feedback may be connected, but it does not specify the relative weight, sequence, or threshold conditions required for these pathways to operate effectively. Further, the framework does not assume that feedback from frontline practices will automatically lead to institutional adaptation, particularly in organisations where regulatory requirements, audit expectations, resource constraints, or hierarchical decision-making may limit responsiveness. The TES Food Safety Framework should therefore be treated as a theoretically informed guide for future empirical examination, rather than as a validated or universally applicable model of Champion effectiveness.

## 7. Feedback Loop: How Practice Reshapes Institutions

If food safety governance is understood simply as a one-way process of policy design, employee execution, and audit inspection, frontline practice runs the risk of becoming passive implementation. However, in reality, production environments are constantly changing, due to staff turnover, equipment ageing, supply chain disruptions, shifting customer demands, and unexpected hazards continuously altering the conditions on which the formal systems were originally built. A management system, therefore, cannot be designed once and remain suitable for all situations indefinitely.

Bromley and Powell [6] argue that institutional failure often arises not because the system itself is wrong, but because it is not being continuously revised or reassessed, thereby allowing formal structures to drift away from their original purpose. For this reason, institutions must shape practices such as hazard identification and risk assessment, but practice must also inform and shape institutions. This is the core function of the feedback loop.

Evidence of a decrease in food safety will frequently first emerge in small deviations during everyday operations. High-reliability systems do not wait for major incidents to occur before taking action; rather, they pay continuous attention to weak signals and minor deviations before significant consequences occur [34,41]. Early, indicators of system failures may include cleaning steps being shortened, deviation reporting being delayed, new employees repeating the same mistakes, or critical control points being routinely bypassed.

The Alliance to Stop Foodborne Illness [38] similarly argues that a mature FSC depends on sustained attention to everyday small problems, rather than only reacting to major events after they occur. Frontline practice is therefore not the endpoint of institutional execution, but the most important source of continuous improvement. The value of the feedback loop lies in bringing these practical signals back into the system, allowing organisations to identify, correct, and learn before risk escalates.

Champions can play an important role in this feedback process, as they not only help institutional expectations become enacted in everyday practice, but also feed frontline realities back into formal systems. Because they understand both institutional logic and frontline conditions, they are often the first person to recognise a misalignment between formal expectations and operational reality. When employees fail to follow a procedure, the problem may not be due to their attitude, but to resource constraints or flaws in the system itself. A procedure that is routinely ignored may be too complex to sustain in practice, while low rates of deviation reporting may reflect an organisational response that discourages people from speaking up.

Pekaar et al. [31] identified an increase in cross-level communication as one of the Champion’s most important functions, allowing management to learn how institutions actually operate under real conditions rather than relying only on formal indicators. The value of a Champion lies not only in them helping institutions enter practice, but also in helping practice re-enter institutions.

If organisations expect Champions to enforce rules but do not allow them to return practical problems to the system, Champions may become trapped between institutions and reality. They are left asking employees to follow rules that may be difficult to enact while defending requirements that the organisation does not sufficiently support.

Such a conundrum can weaken both the Champion’s influence and employee trust in the system. When institutions fail to respond to real conditions, food safety requirements are easily seen as existing for audits rather than for risk reduction, and compliance quickly becomes symbolic. A mature FSC depends on two-way communication, not one-way instruction [17]. Without feedback, institutions become less effective.

The purpose of the feedback loop is therefore not simply to collect opinions, but to convert frontline experience into institutional learning. In this process, Champions not only translate formal expectations downward into practice, but they also translate practice-based signals upward, thereby facilitating organisational adjustment. Repeated workarounds, delayed reporting, shortened cleaning steps, impractical procedures, resource constraints, or recurring deviations can all indicate that formal systems are drifting away from operational reality. When these signals are recognised and reported to the organisation, they can inform SOP revision, training redesign, resource allocation, audit focus, and corrective actions. The feedback loop is therefore central to reducing institutional decoupling as it enables food safety governance to move from static control towards continuous learning, where formal systems are repeatedly tested, adapted, and reconnected with everyday frontline practice.

The proposed TES Food Safety Framework also provides a basis for future empirical examination of the role of the Food Safety Champion. As Food Safety Champions are conceptualised as translational actors rather than simply being motivated individuals, future research should examine how they are trusted, supported, positioned, and able to influence everyday practice. Table 3 outlines possible dimensions through which the Champion construct may be operationalised in future research, including interviews, surveys, comparative case studies, or organisational assessments.

The dimensions outlined in Table 3 are not intended to be used as fixed measurement instruments. Rather, they reinforce the central premise of this paper: Champions become most meaningful when their work connects formal food safety expectations with everyday practice and organisational learning.

## 8. Conclusions

This paper addresses a central question: why do organisations continue to struggle to maintain stable and consistent frontline food safety practices, despite having well established formal food safety management systems, standard operating procedures, and audit mechanisms? It highlights that what matters most is whether organisations have the correct processes and clear expectations through which food safety can be continuously understood, enacted, and sustained under real production conditions.

From this perspective, an FSC is reconceptualised as a process of practice generation rather than a static outcome of institutional compliance. It is not something an organisation possesses; rather, it is continuously enacted through an organisation’s employees’ daily interactions, repeated judgements, and routine behaviours.

This paper defines the Food Safety Champion as being a translational mechanism between institutional expectations and practice enactment. The core function of a Food Safety Champion is not to enforce compliance, but to move food safety from a required task to a stable way of organisational life.

A Champion’s effectiveness does not depend on their commitment, but on the relational structure and organisational conditions in which their role is embedded. Networks, peer trust, and organisational support determine whether the role can be sustained, while feedback loops ensure that institutions remain open to correction through practice rather than drifting into symbolic compliance.

The theoretical contribution of this paper is to introduce the concept of a Food Safety Champion and position it as a translational mechanism between institutional food safety requirements and everyday frontline practice.

As a Perspective grounded in a narrative integrative synthesis, the primary objective of this paper was to provide a conceptual synthesis and establish a theoretical foundation for discussing Food Safety Champions, rather than presenting the data required for empirical validation of the role. The operational dimensions outlined in Table 3 indicate how this framework may be examined in future research through interviews, surveys, comparative case studies, and the interrogation of organisational data across food businesses with different levels of Food Safety Champion implementation. Such empirical work will be important for assessing and providing guidance on how Champion roles can be recognised, supported, and sustained in practice, and how Champions can most effectively influence frontline food safety behaviour under real production pressures.

## Figures and Tables

**Table 1:** Thematic classification of literature informing the Food Safety Champion framework.

Thematic Literature Stream	Indicative Sources	Main Contribution to the Paper	Relevance to the Food Safety Champion Framework
Food Safety Culture as an organisational and behavioural construct	[7,8,9,10,11,12,13,14,15,16,17,18,19,24,25,26]	Defines food safety culture as more than formal compliance, emphasising leadership, employee participation, behavioural consistency, maturity, communication, and organisational context.	Provides the conceptual foundation for explaining why formal food safety systems, HACCP procedures, SOPs, training, and audits do not automatically produce stable frontline behaviour.
Champion roles and value-oriented mechanisms	[27,28,29,30,31,32,33]	Explains how Champions operate through peer influence, role modelling, trust-building, contextual communication, value internalisation, and the translation of organisational goals into locally meaningful practice.	Supports the conceptualisation of Food Safety Champions as translational actors who help connect institutional expectations with situated frontline practice.
Behavioural reinforcement and normalisation of practice	[5,8,18,24,34]	Report how repeated visibility, feedback, modelling, and peer-level reinforcement influence what employees perceive as acceptable, expected, or non-negotiable in everyday work.	Explains how Champions may help prevent unsafe shortcuts from becoming normalised and support the stabilisation of safe food handling behaviours over time.
Peer-based networks, relational support, and learning structures	[32,35,36,37]	Highlight the importance of shared learning, belonging, informal legitimacy, peer trust, and knowledge exchange in sustaining practice beyond formal instruction.	Supports the argument that Champion effectiveness depends not only on individual motivation, but also on relational networks and collective support structures.
Industry and regulatory implementation guidance	[16,17,18,26,38]	Demonstrate how food safety culture is increasingly operationalised through behaviour-oriented assessment, leadership demonstration, employee engagement, and practical implementation tools.	Links the conceptual framework to current industry expectations and shows why Champion roles are practically relevant for strengthening food safety culture in real production environments.
Organisational systems, decoupling, and frontline practice literature	[4,5,6,7,14,39,40,41,42]	Explain how formal structures may become separated from everyday action when institutional requirements are not interpreted, adapted, or reinforced under real working conditions.	Provides the theoretical basis for understanding Food Safety Champions as mechanisms that may support recoupling between formal food safety governance and frontline enactment.

**Table 2:** Functions which distinguish Food Safety Champions from related organisational and implementation roles.

Related Construct	Primary Function	Main Source of Influence	Limitation for Explaining Food Safety Practice	Distinction from the Food Safety Champion Construct
Informal leaders	Influence colleagues through everyday interaction, peer relationships, experience, and social standing rather than formal authority.	Peer trust, practical credibility, social proximity, and visibility in daily work.	Explains peer influence within teams but does not necessarily explain how formal food safety requirements are interpreted, adapted, reinforced, and stabilised under production conditions.	Food Safety Champions may operate informally, but their role is specifically connected to translating food safety expectations into daily routines and reinforcing safe behaviour when formal requirements meet operational pressure.
Change agents	Support organisational change by promoting new practices, encouraging the adoption of and helping implement strategic or procedural initiatives.	Organisational mandate, change-management capability, communication skills, and cross-level influence.	Often focuses on planned change programmes or discrete implementation processes, rather than the continuous enactment of food safety requirements in routine frontline work.	Food Safety Champions are concerned not only with initiating change, but with sustaining food safety expectations as repeated, visible, and socially supported practice over time.
Opinion leaders	Shape attitudes, beliefs, or decisions by being perceived as credible, knowledgeable, or influential within a group.	Expertise, reputation, communication ability, and social influence.	Explains how ideas or messages spread but provides a limited account of information on how institutional requirements become operationally workable under real production constraints.	Food Safety Champions not only influence opinions; they help convert abstract food safety expectations into practical actions and behavioural norms in everyday work.
Safety Champions	Promote safety awareness, encourage compliance, and support safety-related initiatives in organisational settings.	Safety commitment, role modelling, visibility, and perceived dedication to safe practice.	Usually addresses workplace safety broadly and may not capture the specific characteristics of food production, including HACCP controls, hygienic practices, contamination risks, audit pressure, and production-versus-safety tensions.	Food Safety Champions are a food safety-specific form of Champion whose translational work is tied to food safety management systems, HACCP-based expectations, frontline food handling behaviour, and food safety culture maturity.
Boundary spanners	Connect different groups, functions, or organisational levels by enabling communication, coordination, and knowledge transfer.	Cross-functional positioning, relational access, and the ability to translate between groups or organisational levels.	Explains connectivity across boundaries, but does not necessarily address behavioural reinforcement, peer legitimacy, or the stabilisation of safe practice over time.	Food Safety Champions may perform boundary-spanning work, but their distinct contribution lies in linking institutional expectations, frontline enactment, and feedback loops within food safety governance.
Food safety ambassadors	Communicate food safety messages, promote awareness, and represent food safety values within an organisation or community.	Communication, visibility, advocacy, symbolic representation, and positive messaging.	May emphasise awareness or engage in promotional communication without fully explaining how food safety expectations are negotiated, reinforced, and maintained in routine production practice.	Food Safety Champions are not only communicators; they are practice-based translational actors who support meaning making, contextual negotiation, behavioural reinforcement, and organisational learning.
Food Safety Champions	Translate formal food safety expectations into everyday frontline practice and help sustain food safety culture through practical interpretation, behavioural reinforcement, and feedback.	Dual legitimacy: Peer trust and organisational recognition, supported by practical credibility, enabling conditions, networked support, and cross-level communication.	The role may be weakened if treated only as individual enthusiasm without structural support, protected time, role legitimacy, clear boundaries, and feedback channels.	The construct integrates food safety governance, frontline practice, and institutional translation. It explains how formal requirements may become meaningful, workable, reinforced, and continuously improved in food production environments.

**Table 3:** Potential dimensions for the empirical examination of Food Safety Champions.

Dimension	Possible Empirical Indicators	Possible Future Data Sources	Link to the Framework
Peer trust	Whether frontline employees perceive the Champion as being approachable, fair, credible, and familiar with everyday production pressures.	Interviews with frontline staff; staff surveys; focus groups; observation of team interactions.	Supports the Champion’s ability to influence practice through relational legitimacy rather than formal authority.
Practical credibility	Whether the Champion is seen as having relevant food safety knowledge, operational experience, and problem-solving ability.	Interviews; supervisor assessments; peer feedback; observation of issue resolution.	Explains why employees may accept guidance from the Champion during routine work and production pressure.
Organisational legitimacy	Whether the Champion role is recognised, supported, and given clear boundaries by management.	Management interviews; role descriptions; internal documents; training records; resource allocation records.	Indicates whether the Champion has the institutional support needed to sustain translation work.
Behavioural reinforcement activities	Frequency and quality of reminders, role modelling, feedback, correction of deviations, and positive reinforcement of safe practice.	Workplace observation; behavioural checklists; incident or deviation records; staff interviews.	Captures how Champions help stabilise food safety expectations into repeated everyday behaviour.
Translational effectiveness	The extent to which formal food safety requirements are made understandable, workable, and meaningful in frontline routines.	Interviews; case studies; comparison of SOPs with observed practice; examples of rule interpretation during production.	Measures the central mechanism through which Champions connect institutional expectations with practice enactment.
Network position	The Champion’s connection across shifts, departments, functions, and management levels.	Social network mapping; communication records; cross-functional meeting records; interviews.	Shows whether the Champion can move knowledge, concerns, and practical solutions across organisational boundaries.
Feedback-loop contribution	Whether Champions identify recurring barriers, report weak signals, escalate impractical procedures, or contribute to SOP, training, or resource adjustments.	Deviation reports; meeting minutes; corrective action records; audit follow-up documents; management interviews.	Assesses how frontline observations are reported to the formal system and used for institutional adaptation.
Role sustainability	Whether the role is supported by protected time, workload balance, recognition, training, and continuity planning.	Workload review; role descriptions; interviews; staff turnover data; internal recognition or support records.	Evaluates whether the Champion function is sustainable or vulnerable to fatigue, overload, and symbolic implementation.

## Data Availability

No new data were created or analyzed in this study. Data sharing is not applicable to this article.

## Abbreviations

The following abbreviations are used in this manuscript:

FSC	Food Safety Culture
FSMS	Food Safety Management System
HACCP	Hazard Analysis and Critical Control Point
SOP	Standard Operating Procedure
TES	Translation–Enactment–Sustaining

## References

[1] Codex Alimentarius Commission (2020). General Principles of Food Hygiene (CXC 1-1969).

[2] Mortimore S., Wallace C. (2013). HACCP: A Practical Approach.

[3] World Health Organization (2026). WHO Estimates of the Global Burden of Foodborne Diseases 2000–2021.

[4] Pai A.S., Jaiswal S., Jaiswal A.K. (2024). A comprehensive review of food safety culture in the food industry: Leadership, organizational commitment, and multicultural dynamics. Foods.

[5] Meyer J.W., Rowan B. (1977). Institutionalized organizations: Formal structure as myth and ceremony. Am. J. Sociol..

[6] Bromley P., Powell W.W. (2012). From smoke and mirrors to walking the talk: Decoupling in the contemporary world. Acad. Manag. Ann..

[7] Jespersen L., Griffiths M., Maclaurin T., Chapman B., Wallace C.A. (2016). Measurement of food safety culture using survey and maturity profiling tools. Food Control.

[8] Griffith C.J., Livesey K.M., Clayton D.A. (2010). Food safety culture: The evolution of an emerging risk factor?. Br. Food J..

[9] da Cunha D.T., Stedefeldt E., Luning P.A., Prates C.B., Zanin L.M. (2025). Food safety culture as a behavioural phenomenon shaping food safety. Curr. Opin. Food Sci..

[10] Spagnoli P., Vlerick P., Pareyn K., Foubert P., Jacxsens L. (2025). Portfolio of interventions to mature human organizational dimensions of food safety culture in food businesses. Food Control.

[11] Miebach B. (2017). Handbuch Human Resource Management: Das Individuum und Seine Potentiale für die Organisation.

[12] De Boeck E., Jacxsens L., Mortier A.V., Vlerick P. (2018). Quantitative study of food safety climate in Belgian food processing companies in view of their organizational characteristics. Food Control.

[13] Wang K., Mirosa M., Hou Y., Bremer P. (2026). Measuring food safety culture: A systematic review of questionnaire dimensions and validation practices. Compr. Rev. Food Sci. Food Saf..

[14] Wilcock A., Ball B., Fajumo A. (2011). Effective implementation of food safety initiatives: Managers’, food safety coordinators’ and production workers’ perspectives. Food Control.

[15] Zanin L.M., Stedefeldt E., Luning P.A. (2021). The evolvement of food safety culture assessment: A mixed-methods systematic review. Trends Food Sci. Technol..

[16] BRCGS (2023). Food Safety Culture Excellence: Driving Continuous Improvement in Food Safety Culture (FSCE Brochure).

[17] Food Standards Australia New Zealand (2025). Shaping Food Safety Culture.

[18] Jespersen L., Choiniere C., Ronke M.E., Coffman V. (2022). Building a Coalition of Food Safety Champions.

[19] Frankish E.J., McAlpine G., Mahoney D., Oladele B., Luning P.A., Ross T., Bowman J.P., Bozkurt H. (2021). Review article: Food safety culture from the perspective of the Australian horticulture industry. Trends Food Sci. Technol..

[20] Arksey H., O’Malley L. (2005). Scoping Studies: Towards a Methodological Framework. Int. J. Soc. Res. Methodol..

[21] Levac D., Colquhoun H., O’Brien K.K. (2010). Scoping Studies: Advancing the Methodology. Implement. Sci..

[22] Munn Z., Peters M.D.J., Stern C., Tufanaru C., McArthur A., Aromataris E. (2018). Systematic Review or Scoping Review? Guidance for Authors When Choosing between a Systematic or Scoping Review Approach. BMC Med. Res. Methodol..

[23] Popay J., Roberts H., Sowden A., Petticrew M., Arai L., Rodgers M., Britten N., Roen K., Duffy S. (2006). Guidance on the Conduct of Narrative Synthesis in Systematic Reviews: A Product from the ESRC Methods Programme.

[24] Wang K., Mirosa M., Hou Y., Bremer P. (2025). Advancing food safety behavior with AI: Innovations and opportunities in the food manufacturing sector. Trends Food Sci. Technol..

[25] Li Q., Zhang B., Dai Y., Zhang H., Liu P. (2024). Progress analysis of international food safety culture construction and its enlightenment to China. Sci. Technol. Food Ind..

[26] Baeza R., Kluse C. (2024). Measuring food safety culture in food manufacturing through different metrics. J. Manag. Eng. Integr..

[27] Amaya M., Melnyk B., Buffington B., Battista L. (2017). Workplace wellness champions: Lessons learned and implications for future programming. Build. Healthy Acad. Communities J..

[28] Cave D., Abbey K., Capra S. (2021). Food and nutrition champions in residential aged care homes are key for sustainable systems change within foodservices: Results from a qualitative study of stakeholders. Nutrients.

[29] Solitander N., Fougère M., Sobczak A., Herlin H. (2012). We are the champions: Organizational learning and change for responsible management education. J. Manag. Educ..

[30] May B. (2017). How to Make Your Company a Recognised Sustainability Champion.

[31] Pekaar K.A., Demerouti E., van Gool P.J.R. (2024). Sustainability champions: A proactive perspective on the inter-organizational job design dynamics of sustainability implementation. Organ. Psychol. Rev..

[32] Alm K., Hultman J. (2023). Sustainability ambassadorship: The role of the store manager in development of in-store sustainability communication. Int. Rev. Retail Distrib. Consum. Res..

[33] Apid A.J., Teng-Calleja M. (2025). Multilevel influence of sustainability leaders in business systems. J. Manag. Glob. Sustain..

[34] Reason J. (2000). Human error: Models and management. BMJ.

[35] Sullivan J.M. (2017). Creating Employee Champions: How to Drive Business Success through Sustainability Engagement Training.

[36] Francis E., Hogentogler R., Hoke A., Buckley J., Hwang G., Lehman E., Kraschnewski J.L. (2019). The Healthy Champions program in Pennsylvania schools: Assessment, awareness, and improvement of school wellness. Prev. Med. Rep..

[37] Yung K., Neathway C. (2020). Community champions for safe, sustainable, traditional food systems. Curr. Dev. Nutr..

[38] Alliance to Stop Foodborne Illness (2023). Food Safety Culture Toolkit.

[39] Nyarugwe S.P., Linnemann A.R., Hofstede G.J., Fogliano V., Luning P.A. (2016). Determinants for conducting food safety culture research. Trends Food Sci. Technol..

[40] McLeod R.W., Al Hashmi W.S.G. (2021). The awareness of risk, complacency, and the normalization of deviance. Process Safety Management and Human Factors.

[41] Weick K.E., Sutcliffe K.M. (2007). Managing the Unexpected: Resilient Performance in an Age of Uncertainty.

[42] Lee J.C., Neonaki M., Alexopoulos A., Varzakas T. (2023). Case Studies of Small-Medium Food Enterprises around the World: Major Constraints and Benefits from the Implementation of Food Safety Management Systems. Foods.

[43] Czarniawska-Joerges B., Sevón G. (1996). Translating Organizational Change.

[44] Morris T., Lancaster Z. (2006). Translating Management Ideas. Organ. Stud..

[45] Sahlin K., Wedlin L., Greenwood R., Oliver C., Suddaby R., Sahlin K. (2008). Circulating Ideas: Imitation, Translation and Editing. The SAGE Handbook of Organizational Institutionalism.

[46] Lawrence T.B., Suddaby R., Clegg S.R., Hardy C., Lawrence T.B., Nord W.R. (2006). Institutions and Institutional Work. The SAGE Handbook of Organization Studies.

[47] Yiannas F. (2008). Food Safety Culture: Creating a Behavior-Based Food Safety Management System.

[48] Taylor J. (2011). An exploration of food safety culture in a multi-cultural environment: Next steps?. Worldw. Hosp. Tour. Themes.

[49] De Clercq D., Pereira R. (2023). Proactive champions: How personal and organizational resources enable proactive personalities to become idea champions. J. Soc. Psychol..

[50] Shea C.M. (2021). A conceptual model to guide research on the activities and effects of innovation champions. Implement. Res. Pract..

[51] Fathi L., Young C., Cleary A., Porter D., Taylor D., Dick M., Morrison C., Chai L.K., Nethery Z., Cacavas K. (2025). From “What’s this?” to “I grew it!”: Evaluation of a school nutrition program in Queensland, Australia. Health Promot. J. Austr..

[52] Battistella C., Annarelli A., Nonino F. (2015). Exploring the impact of organizational and working models, incentives and collaboration strategies on innovation development in online communities of practices. Proceedings of the International Conference on Knowledge Management and Information Sharing.

[53] Workforce Development Ltd., G&H Training Ltd., FutureCol (2018). Communities of Practice Training Workbook: Developing Communities of Practice as a Pedagogy Support Mechanism for Teaching Teams in the New Zealand PTE Environment.

[54] Liu X., Mirosa M., Bremer P. (2026). A Guide for Food Safety Culture Champions to Recognise and Support Frontline Food Safety Champions.

